# Translocation of silica nanospheres through giant unilamellar vesicles (GUVs) induced by a high frequency electromagnetic field

**DOI:** 10.1039/d1ra05459g

**Published:** 2021-09-23

**Authors:** Palalle G. Tharushi Perera, Nevena Todorova, Zoltan Vilagosh, Olha Bazaka, The Hong Phong Nguyen, Kateryna Bazaka, Russell J. Crawford, Rodney J. Croft, Irene Yarovsky, Elena P. Ivanova

**Affiliations:** School of Science, RMIT University PO Box 2476 Melbourne VIC 3001 Australia elena.ivanova@rmit.edu.au; Faculty Science, Engineering and Technology, Swinburne University of Technology PO Box 218 Hawthorn VIC 3122 Australia; School of Engineering, RMIT University PO Box 2476 Melbourne VIC 3001 Australia; Faculty of Applied Sciences, Ton Duc Thang University Ho Chi Minh City Vietnam; School of Engineering, College of Engineering and Computer Science, The Australian National University Canberra ACT 2600 Australia; School of Psychology, Illawarra Health and Medical Research Institute, University of Wollongong Wollongong NSW 2522 Australia

## Abstract

Membrane model systems capable of mimicking live cell membranes were used for the first time in studying the effects arising from electromagnetic fields (EMFs) of 18 GHz where membrane permeability was observed following exposure. A present lack of understanding of the mechanisms that drive such a rapid change in membrane permeabilization as well as any structural or dynamic changes imparted on biomolecules affected by high-frequency electromagnetic irradiation limits the use of 18 GHz EMFs in biomedical applications. A phospholipid, 1,2-dioleoyl-*sn*-glycero-3-phosphocholine (DOPC) labelled with a fluorescent marker 1,2-dioleoyl-*sn*-glycero-3-phosphoethanolamine-*N*-(lissamine rhodamine B sulfonyl) (rhodamine-DOPE) was used in constructing the giant unilamellar vesicles (GUVs). After three cycles of exposure, enhanced membrane permeability was observed by the internalisation of hydrophilic silica nanospheres of 23.5 nm and their clusters. All-atom molecular dynamics simulations of 1-palmitoyl-2-oleoyl-*sn*-glycero-3-phosphocholine (POPC) membranes exposed to high frequency electric fields of different field strengths showed that within the simulation timeframe only extremely high strength fields were able to cause an increase in the interfacial water dynamics characterized by water dipole realignments. However, a lower strength, high frequency EMF induced changes of the water hydrogen bond network, which may contribute to the mechanisms that facilitate membrane permeabilization in a longer timeframe.

## Abstract 2

Membrane model systems capable of mimicking live cell membranes were used for the first time in studying the effects arising from electromagnetic fields (EMFs) of 18 GHz where membrane permeability was observed following exposure.

## Graphical abstract

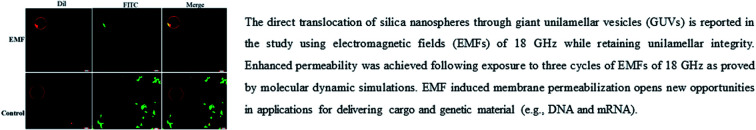

## 1. Introduction

A highly controlled exposure of living organisms to high frequency electromagnetic fields (EMF) of low field strength has emerged as a promising technique to efficiently induce temporary permeability with a high level of post-treatment viability in a wide variety of cells. To this effect, a wide range of EMFs have been trialed, with an EMF of 18 GHz showing a highly favourable combination of treatment efficiency, cell viability and induced biochemical changes. First demonstrated in *Escherichia coli* with the help of dextran probes (15.9 nm),^1–3^ the ability of a brief 18 GHz treatment to trigger reversible permeabilization in cellular membranes was later demonstrated in a large range of morphologically and structurally distinct microorganisms, including Gram positive cocci,^4^*Planococcus maritimus* KMM 3738, *Staphylococcus aureus* CIP65.8^T^, *S. aureus* ATCC 25923 and *S. epidermidis* ATCC 14990^T^, as well as eukaryotic cells, such as *Saccharomyces cerevisiae* ATCC 287 and pheochromocytoma cells.^5^ Transmission Electron Microscopy (TEM) results revealed that the EMF-treated cells were able to internalise nanospheres of 23.5 nm and 46.5 nm in diameter. The degree of internalisation was found to be variable, most likely due to differences in the cell wall structure of exposed organisms.^4^ Successful induction of permeabilization across all types of prokaryotic and eukaryotic cells irradiated with 18 GHz EMFs suggests certain universal processes that take place in water and/or at the interface under these irradiation conditions.

Currently there is a lack of understanding of the mechanisms that explain membrane permeabilization induced through exposure to 18 GHz EMFs. Indeed, the complexity of prokaryotic and eukaryotic cells and biological systems makes identification of individual physical, chemical and biological events challenging. To aid with such an investigation, a wide range of *in vitro* models that mimic specific cellular components or processes in cells have been designed to understand how they are affected as a result of exposure to a specific stimulus, in our case, the polarizing electromagnetic oscillations. Among available systems, the resemblance of liposomes to biological membranes makes them an excellent tool for the study of processes that take place at the cellular membrane.

Therefore, the aim of this study was to: (i) study the effects of 18 GHz EMF exposure on the permeabilization and transport function of cellular membranes, using a model lipid bilayer system; (ii) study the potential use of 18 GHz exposure as a remote physical trigger for liposome permeabilization for site-specific release of cargo in drug and gene delivery systems. The experimental studies were complemented by all-atom molecular dynamics simulations to provide an atomistic insight into the EMF influences on the structure and dynamics of the membrane in a model physiological solution at a space/time resolution not yet achievable by experimental methods. Computational studies have proven to be a useful technique for ranking relative field effects on biomolecular systems as well as understanding molecular mechanisms of such effects by directly observing the real time changes to 3D structure in all-atom details at a shorter timeframe than that accessible to experimental observations.^6–13^

## 2. Materials and methods

### 2.1. Lipids

The lipids were purchased from Avanti Polar Lipids (Alabaster, AL) in the liquid form, dissolved in chloroform. The lipid solutions were stored at −20 °C when not in use. 1,2-dioleoyl-*sn*-glycero-3-phosphocholine (DOPC) was used with a fluorescent marker 1,2-dioleoyl-*sn*-glycero-3-phosphoethanolamine-*N*-(lissamine rhodamine B sulfonyl) (rhodamine–DOPE) in a 0.2 mol% mixture.

### 2.2. Silica nanospheres

Fluorescent silica nanospheres with a diameter of 23.5 ± 0.2 nm (FITC) (Corpuscular, Cold Spring, NY, USA) were used to investigate the membrane permeability of the samples that were exposed to EMF treatment.

### 2.3. PVA–VP(poly(vinyl alcohol)–vinyl pyrrolidone)

The mesoporous hydrogels were synthesised through free radical polymerisation of linear poly(vinyl alcohol) (PVA) using the cross linker n-vinyl pyrrolidone (VP). High molecular weight 125 kDa PVA (abbreviated as H-PVA) was used in synthesising the gel. Ceric ammonium nitrate (CAN) was used to initiate the chain reaction leading to the formation of more free radicals. Aqueous PVA solution (5 wt%) was prepared by dissolving PVA in H_2_O at 70 °C under constant magnetic stirring. The solution was subsequently cooled down to room temperature. An appropriate weight of n-vinyl pyrrolidone (VP) monomer (0.1 mL) was added into the PVA solution under magnetic stirring at a maintained temperature of 40 °C. Ceric ammonium nitrate (CAN) was added as an initiator (0.8 mL) of the free-radicle polymerization reaction. The entire system was purged continuously with N_2_ gas for 3 hours to remove the presence of any oxygen bubbles. The resulting homogenous polymer solutions were cast on a glass side (76 mm × 26 mm × 1 mm) for the synthesis of GUVs.

### 2.4. Construction of GUVS

**Fig. 1:**
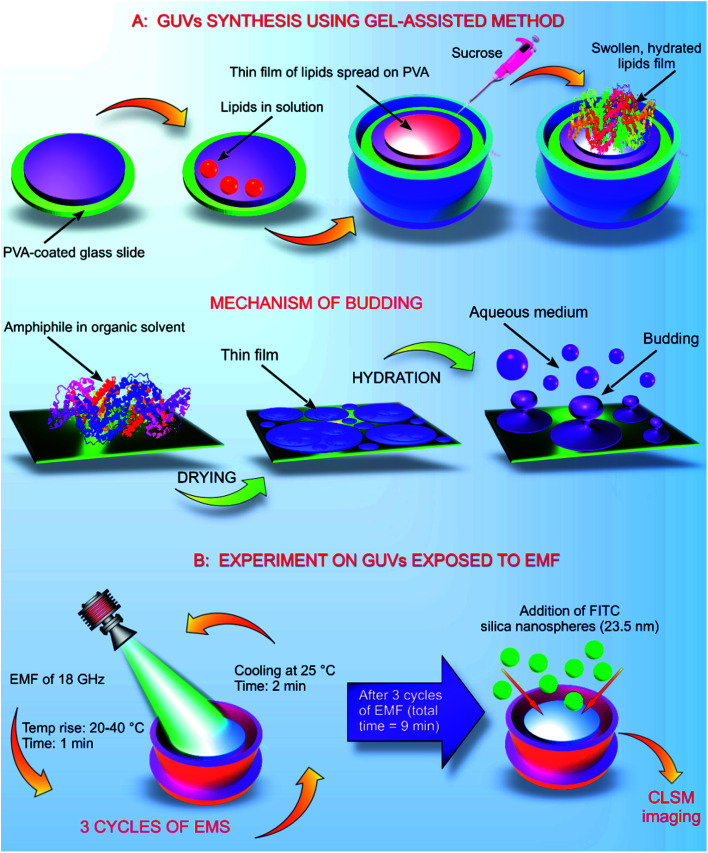
Synthesis of GUVs and their repeated exposure to EMF of 18 GHz. (A) GUVs synthesis using the gel assisted method. A thin layer of hydrogel was spread on the ozone cleaned glass slide. Lipids were placed on the hydrogel and placed under a vacuum. Placing the lipids inside the vacuum helped evaporate the solvent. The lipids were originally present in a solvent (chloroform). Addition of an aqueous solvent, 280 mM sucrose helped to hydrate the lipid film, generating liposomes as illustrated. (B) Schematic representation of the cycles of EMF exposure. The first stage of the EMF treatment increased the temperature of the GUVs to 40 °C. After the treatment (1 min), the GUVs in solution were allowed to cool down at 25 °C for 2 min before the next exposure. After the three EMF treatments, the GUVs were imaged after addition of the silica nanospheres.

The GUVs were constructed using the gel assisted method as described elsewhere.^14^ In brief, glass slides (25 mm × 75 mm × 1 mm) were cleaned with chloroform, toluene, acetone and 100% ethanol. After the final wash of ethanol, the glass slides were dried using an ozone cleaner for 60 min. A thin layer of high molecular weight PVA–VP was created on the glass slide using 50 μL of the hydrogel, the PVA film was dried at 80 °C for 30 min. On to the dry PVA film, 50 μL of the stained lipid in chloroform was added and kept under a vacuum for 30 min. The O-rings used in the process were cleaned using toluene, acetone and 100% ethanol. After evaporation of chloroform, the O-ring was placed on the glass slide covering the area containing the stained lipid. Into the O-ring, 1 mL of 280 mM sucrose was added and kept for 15 min. From the mixture, 0.5 mL of sucrose and the lipid were transferred into an Eppendorf tube. Another 0.5 mL of 280 mM glucose was added, and the volume adjusted to 1 mL (Fig. 1).

### 2.5. Exposure of GUVs to EMF of 18 GHz

Exposure of the GUVs to a microwave field was carried out according to the previously developed procedure as described elsewhere.^4,5^ The increase in temperature in the presence of EMF radiation was monitored throughout the duration of the treatment cycle. GUVs prepared in a 280 mM aqueous sugar solution were exposed to EMFs in 60 s-long cycles, which allowed for the temperature to be maintained below 40 °C. The MW apparatus (Lambda Technologies Vari-Wave Model LT 1500) used in the study has an option of varying the frequency range from 5–18 GHz. The frequency was adjusted to a fixed frequency of 18 GHz at a power of 17 W, as described elsewhere.^4,15^ The LT 1500 instrument is capable of delivering controlled EMF as the frequency and the power can be adjusted accordingly. The frequency delivered by the LT 1500 is well above a household microwave which is 2.45 GHz. The Greiner dish (35 mm diameter, Griener Bio One, Frickenhausen, Germany) with the sample was placed on the ceramic pedestal (Pacific Ceramics, Sunnyvale, CA, USA, *ε*′ = 160, loss tangent < 10^−3^) on the hotspot free location, identified by electric field modelling using CST Microwave Studio 3D Electromagnetic Simulation Software (CST MWS) (CST of America, Framingham, MA, USA) and experimental temperature measurements. The temperature rise in the GUVs suspension was monitored using a built-in temperature probe, a Luxtron Fiber Optic Temperature Unit (LFOTU) (LumaSense Technologies, Santa Clara, CA, USA). After the MW treatment, the sample was cooled down to 25 °C for 2 min. The microwave chamber was cooled using ice packs to bring the temperature to 25 °C. The sample was exposed to three cycles (60 s; 2 min cooling) of MW radiation while keeping all the other environmental factors constant.

### 2.6. Dosimetry

The dose of EMF that was delivered to the GUVs samples was calculated as described elsewhere.^2,16^ The rate of electromagnetic energy that is delivered to the sample is expressed as the specific absorption rate (SAR, W kg^−1^), as described elsewhere.^4,16^ In brief, the SAR was measured under the assumption that all the absorbed field energy was transformed into heat, with any heat dissipation being disregarded. Under these circumstances, SAR can be expressed as:SAR = (*σE*^2^)/*ρ*where *σ* is the sample electric conductivity, *E* is the electric field in the sample and *ρ* is the sample density.

Water at 18 GHz has a temperature dependent absorption coefficient of 17–22 cm^−1^,^17^ resulting in a perpetration depth of 0.58 to 0.45 mm. Lipids have an absorption coefficient of ∼0.1–2 cm^−1^ at 18 GHz. Given that the sample composition is mostly water, a 0.6 mm depth sample Petri dish will have a SAR reduced by a factor of ∼3–3.5 at the base of the dish when compared to its surface. The electric conductivity of water at 18 GHz is ∼30 S m^−1^,^17^ and for lipids it is ∼0.02 to 0.6 S m^−1^.^18,19^ Assuming a density for water of 1000 kg m^−3^ and lipids of 950 kg m^−3^ (ref. 20) and given the stated SAR near the surface of the sample of 5 kW kg^−1^ in the experiment^5^ is for the water component, SAR (lipid) is ∼1.0 to 2.2 W kg^−1^, a reduction of a factor of 3000 to 5000 over the SAR for water.

The instantaneous temperature rise was calculated using the equation:^21^d*T* = d*t* SAR/*C*where d*T* is the temperature rise, d*t* is the time and *C* is the specific heat capacity.

The maximal rate would have to be specified for the top of the Petri dish. An infinitesimal d*t* gives an instantaneous temperature rise. For the water-based solution, assuming a heat capacity for water of *C* = 4.18 kJ kg^−1^ C^−1^ (ref. 21) and a SAR 5 kW kg^−1^, gives a d*T* of 1.2 °C s^−1^. Given that the sample heats by 20 °C over 60 s, the estimate is in line with expectations, once the losses to the surrounding structures are considered. The GUVs near the top of the Petri dish, given a heat capacity for lipids of *C* = 1.8 to 2.7 kJ kg^−1^ C^−1^,^22^ a SAR 1.0 to 2.2 W kg^−1^, gives a dT of 0.0006 to 0.0012 °C s^−1^.

### 2.7. Molecular dynamics simulations of POPC membrane exposure to EMFs

To explore the impact of high-frequency EMFs on a membrane model, the CHARMM-GUI building platform^23^ was used to construct the POPC bilayer model comprising 200 lipids per leaflet. The system was solvated with approximately 50 water molecules per lipid and physiological salt concentration of 150 mM NaCl. All simulations were performed using the GROMACS 5.1.2 simulation package^24^ in conjunction with the CHARMM36m force-field.^25^ During the initial relaxation phase under ambient conditions (303.15 K, 1 bar, no EMF), the membrane system was subjected to energy minimization using the steepest-descent algorithm and restrained molecular dynamics simulations, as prescribed by the CHARMM-GUI input generator.^26^ Following the relaxation stage, un-restrained production simulations were performed under ambient and electric field conditions (details below). The Particle Mesh Ewald (PME) method^27^ was used to evaluate the long range electrostatic interactions, with a cut-off of 10 Å for the direct space sum, and a spacing of 1.2 Å for the FFT (Fast Fourier transform) grid. The van der Waals interactions were truncated at 10 Å. The LINCS algorithm was used to constrain all hydrogen bond lengths, which enabled an integration time step of 2 fs to be used.^28^ The MD simulations were performed under semi-isotropic ensemble with constant particle number, pressure, and temperature (NPT), utilizing the Nose–Hoover thermostat^29^ and Parrinello-Rahman barostat^30^ to maintain temperature at 303.15 K and pressure of 1 bar.

To emulate membrane exposure to electromagnetic fields non-equilibrium molecular dynamics simulations were employed which included an external electric force added to the intermolecular interaction forces used in the Newtonian equation. Newton's second law becomes *m*_i_***r̈***_i_ = ***f***_i_ + *q*_i_***E***(*t*) where the force ***f***_*i*_, is due to the forcefield determined interactions between all particles, *q*_i_***E*** is the electric field force applied to each partial charge i with charge *q*_i_.^31^ The oscillating nature of EMF was modeled using *E* = *E*_0_ cos (*ωt*), where frequency was set to 18 GHz. For a more detailed theoretical background and other examples of applications of EM fields in computational simulations see a comprehensive review.^10^

To equilibrate the POPC membrane, ambient (no external field) simulations were initially performed for 200 ns. Subsequent non-equilibrium simulations were conducted with application of external high frequency (18 GHz) electromagnetic fields of different strengths. The effective RMS field strengths investigated, 
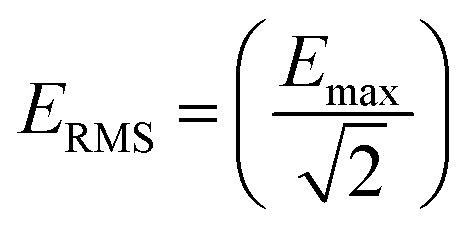
 with the following peak (and respective RMS) field strengths were 0.3 V nm^−1^ (0.21 V nm_rms_^−1^), 0.003 V nm^−1^ (0.0021 V nm_rms_^−1^), 0.000003 V nm^−1^ (0.0000021 V nm_rms_^−1^). The electric field was applied in the *z*-direction, perpendicular to the membrane plane. It is worth noting that field strengths applied in this study are several orders of magnitude larger than those applied experimentally. Previous simulation studies have shown it is necessary to use field strengths of the order of 1 V nm^−1^ to observe tangible effects within the limited time scales amenable to simulation.^6,10^ With this in mind, this work provides a systematic comparison of EF simulations in the intermediate to low strength range (*i.e*., 0.3 to 3 × 10^−6^ V nm^−1^) over hundreds of nanoseconds, which has not been previously reported. Such approach allows for ranking of the field strength dependent effects as well as identifying field induced molecular mechanisms by directly observing the real time changes to molecular structure at different field strengths.^11,13,32^ All field condition simulations were performed for 200 ns each. Monitoring several basic system properties, such as total energy fluctuations and mean square displacements of the lipid phosphorus atoms in the individual trajectories showed that the simulations had achieved a steady state within 150 ns. Hence, the last 50 ns of the simulation trajectory, which equates to 900 cycles of applied electric field, for each field condition was used for analysis.

The effects of electric field exposure on the structural integrity of the POPC membrane were quantified by measuring the lipid mean square displacements, area per lipid distribution, deuterium order parameters and mass density profiles of individual system components. The spatial arrangements of water molecules and ions at the membrane surface and within the leaflets were studied through atomic radial distribution functions and dipole orientation. The hydrogen bonding of water to the lipid and individual lipid components were investigated by calculating the average number of hydrogen bonds formed and their lifetimes.

## 3. Results and discussion

High frequency (18 GHz) EMF radiation treatment offers a promising means for inducing transient permeability in a wide range of cells, and as such can be used in drug delivery and gene therapy applications in medicine and biotechnology, and for the study of fundamental biochemical processes within cells. Due to its favorable combination of high efficiency with respect to inducing permeabilization and lower field strengths that afford higher cell viability rates, 18 GHz EMF can potentially be integrated into lab-on-chip devices as a means for in-depth investigation of transport of exogenous molecules into cells (*e.g*., study of molecular interactions with intracellular components) or efficient extraction of biomolecular components out of cells.^33^ However, for practical realization of these applications, it is important to understand the mechanisms by which EMF induces permeabilization, which is not trivial due to the multitude of interrelated biological, chemical and physical responses that EMF could trigger within cells. For this purpose, in this study, a simple well-defined model membrane system made up of DOPC–RhodPE was chosen to visualise the potential physico-chemical responses of the lipid bilayers to EMFs of high frequency. The main advantages of this single layer lipid vesicle membrane system include ease of preparation and handling and close resemblance to the basic compartment structure of biological cells.^34,35^ The GUVs used in this study are composed of conventional phospholipids with one hydrophilic head group attached to two lipophilic chains^35^ that are directed towards the aqueous media and hydrophobic fatty acid chains forming the interior layer of the bilayer.^35,36^ The formation of liposomes occurred in less than 5 min after the aqueous sugar solution was added to the partially ordered stack of lipids due to spontaneous swelling of the dry lipid films, with the hydration process left undisturbed throughout the procedure.

**Fig. 2:**
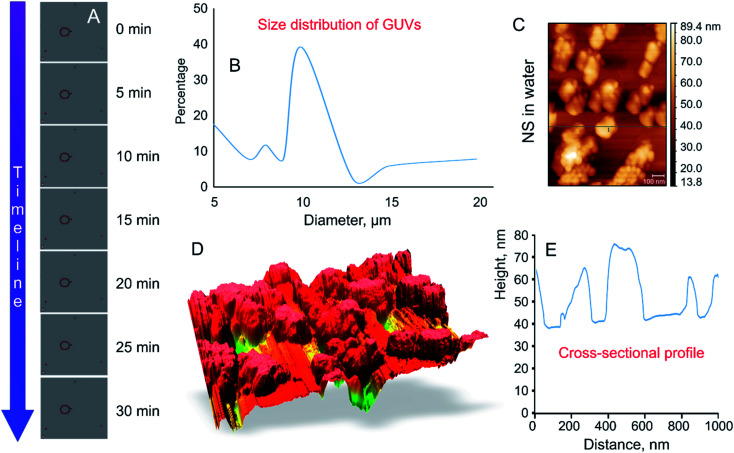
Size distribution and stability of GUVs and nanospheres. (A) Time lapse images of GUVs constructed using the gel assisted method, in suspension. Images were captured continuously for a period of 30 min at 0, 5, 10, 15, 20, 25, 30 min. The GUVs remained stable in solution before exposure to EMF radiation. Scale bar 5 μm. (B) The size distribution of GUVs. The diameter of the liposomes synthesised using the gel assisted method ranged from 5–20 μm (C–E) Atomic force microscopy of the nanosphere clusters in water and corresponding cross-sectional profile (right) indicating the height and width of the nanosphere cluster. The nanospheres were vortexed for one min in order to keep the experimental conditions consistent. The nanospheres tend to exist in clusters of 10 or more nanospheres (200–1000 nm).

The size of thus-prepared liposomes ranged from 5 to 20 μm (Fig. 2). Time-lapse confocal microscopy over a period of 30 min confirmed excellent stability of thus-constructed liposomes. Examination of the images captured at 5 min intervals show that the liposomes preserved their structural integrity and remain in solution without bursting (Fig. 2A). The highest fraction (39.8%) of the GUVs had a diameter of 10 μm, followed by liposomes with a size of 5 μm (17.6%). Presence of liposomes with a size of 10–20 μm varied in accordance with the size distribution curve (Fig. 2B). To determine whether agglomeration indeed took place, atomic force microscopy was used to determine the size of the nanosphere clusters in solution. The AFM images obtained from vortexed (1 min) nanosphere samples in water indicate that the average height of a cluster was approximately 30 nm whereas the diameter ranged from 200 to 1000 nm (Fig. 2C and D). Considering the silicon particles used in this study are spherical, the cross-sectional profile confirmed that the nanoparticles largely exist as clusters, the average size of which is similar to that incorporated into the lipid membranes as visualised by confocal microscopy. Dynamic light scattering results of the sonicated solution of nanospheres reveal that they are mostly present in smaller clusters of ∼63 nm.^16^

**Fig. 3:**
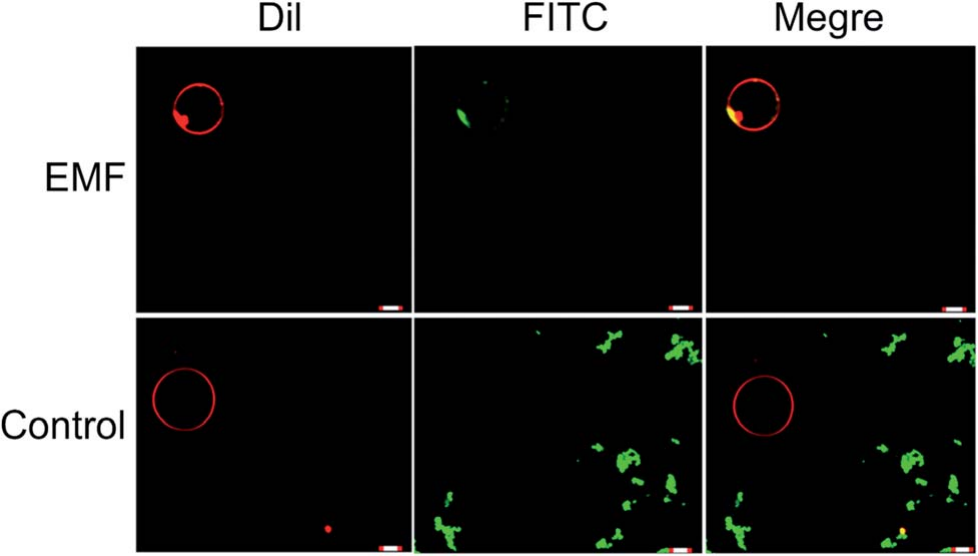
EMF induced membrane permeability of GUVs. Confocal laser scanning micrographs showing the GUVs internalising nanospheres following EMF exposure. The original solution containing the nanospheres were sonicated before addition GUVs. The non-treated GUVs were used as the control, no nanosphere uptake was visualised, while the EMF treated GUVs (top row) were able to internalise nanospheres (existing as clumps). The presence of the green signal (FITC) overlapping with the red signal (Dil) when merged confirms that the nanospheres are present bound to the GUVs. Membrane permeability was not detected in the untreated GUVs as there was no overlapping of the FITC and Dil channels.

In a manner similar to that observed in microbial and mammalian cells^4,16^ treatment of liposomes with EMFs of 18 GHz induced membrane permeabilization in the lipid bilayer. The membrane permeability was demonstrated by treatment-induced internalisation of hydrophilic silica nanospheres of 23.5 nm in diameter and their clusters. This type of nanospheres was purposefully selected for this study since their hydrophilic nature renders their passive transport across the hydrophobic lipid bilayer challenging. Prior to their addition to EMF-treated GUVs, the nanospheres were either sonicated and/or vortexed in order to re-disperse the particles and prevent their aggregation. The internalisation of the nanospheres and their clusters by the liposomes was then visualised using confocal laser scanning microscopy, with the typical images shown in Fig. 3.

All-atom classical molecular dynamics simulations were used to explore the initial (short time post-exposure) effects of high frequency (18 GHz) electromagnetic fields of different field strengths on the atomistic structure and dynamics of the POPC membrane and surrounding solution (water and ions). The model membrane was simulated at ambient (no field) conditions to obtain equilibrium properties for benchmarking and then exposed to 18 GHz electric field of 0.21, 0.0021 and 0.0000021 V nm_rms_^−1^ field strength. The influence of high frequency electric field exposure on the lateral diffusion (dynamics) of lipids was quantified by calculating the mean square displacements of the phosphorus atoms under ambient and electric field conditions.

**Fig. 4:**
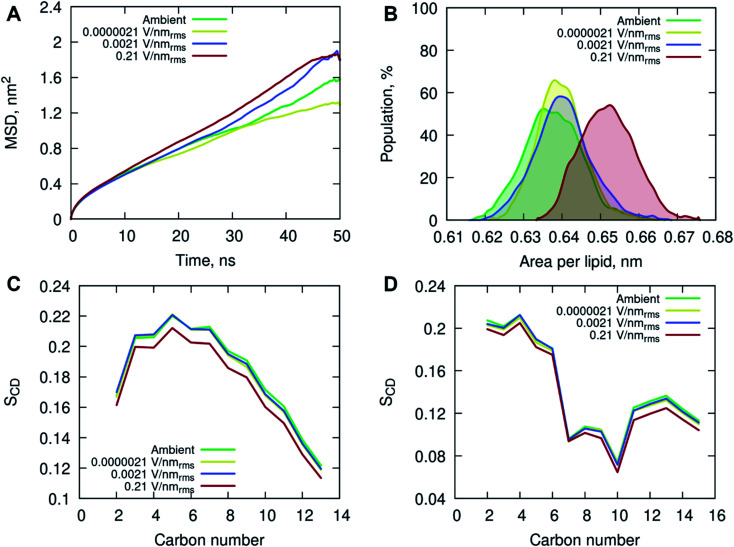
(A) Mean square displacement of the phosphorus (P) atoms in the POPC head group, and (B) area per lipid (nm) distribution determined by taking the unit cell area divided by the number of lipids per leaflet for each frame in the final 50 ns of simulation. Deuterium order parameter *S*_CD_ for saturated *sn*-1 (C) and unsaturated *sn*-2 (D) acyl chains of POPC lipids at different simulated conditions. The properties are depicted in different colors for the following simulated conditions: ambient (green), 0.0000021 V nm_rms_^−1^ (yellow), 0.0021 V nm_rms_^−1^ (blue) and 0.21 V nm_rms_^−1^ (red) electric field strengths.

The MD simulations indicated an increase in long-range lateral diffusion rate of lipids with increasing field strength (Fig. 4A). The high strength (0.21 V nm_rms_^−1^) electric field affected the structural properties of the POPC membrane as indicated by a significant increase in the area per lipid compared to the ambient and lower field strength simulations (Fig. 4B). The ambient and lower field strength conditions produced similar area per lipid distributions, yet the zero-field simulations produced structures with the lowest average area per lipid. The average membrane thickness decreased with increasing field strength, 3.915 ± 0.038 nm (ambient) → 3.905 ± 0.030 nm (0.0000021 V nm_rms_^−1^) → 3.899 ± 0.036 nm (0.0021 V nm_rms_^−1^) → 3.846 ± 0.036 nm (0.21 V nm_rms_^−1^), suggesting the increasing field strength induced a slight compression of the membrane leaflets and overall thinning of the membrane. Fluorescence correlation spectroscopy combined with MD simulations investigated the influence of sodium chloride on a pure POPC lipid bilayer, and showed that an increase in concentration (up to 220 mM) of NaCl contributed to a decrease in lipid diffusion and area per lipid, and consequently an increase in the membrane thickness.^37^ The contrasting results observed in this study, conducted with 150 mM NaCl concentration, indicate that the applied high energy electric fields have a direct effect on the membrane behavior.

The increased lipid dynamics and diminished lipid packing density seen under the applied high electric field conditions may provide a favorable environment for membrane penetration. The area per lipid is highly sensitive to the attractive interactions between the lipid head groups and the dispersion interactions within the non-polar hydrocarbon tails. An increase in the area per lipid can be an indication of an enhanced fluidity of a lipid membrane, suggesting a more disordered hydrophobic membrane core. This is evident from the calculated deuterium order parameter S_CD_ of the hydrocarbon lipid chains seen in Fig. 4C and D. The order parameter S_CD_ was calculated separately for each hydrocarbon group in the POPC acyl chains as *S*_CD_ = 3/2〈cos^2^ *θ*〉 − 1/2, where *θ* is the angle between a CD-bond and the bilayer normal. The deuterium order parameter results show a small increase in disorder in both saturated *sn*-1 and unsaturated *sn*-2 acyl chains of POPC under the applied high electric field conditions. The simulations at lower field strengths exhibited commensurate hydrocarbon chain order to that seen in the ambient (zero field) conditions.

**Fig. 5:**
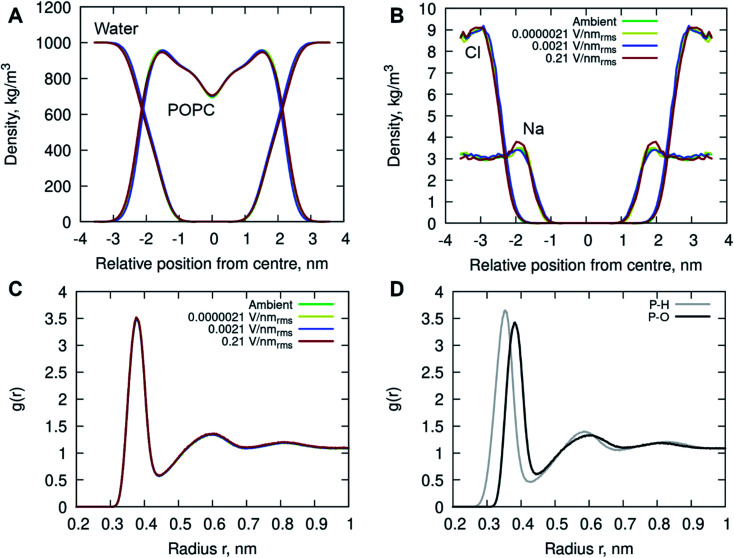
The (symmetrized) mass density profiles of the individual system components, (A) POPC lipids and water molecules, (B) sodium ions (Na^+^) and chloride ions (Cl^−^) for all simulations. (C) Radial distribution functions of the centre of mass (COM) of water relative to the phosphorous (P) atom in the lipid head group. (D) The RDFs of water hydrogens (H, grey) and oxygen (O, black) to the phosphorous atom. The profiles are depicted in different colors identifying the following simulated conditions: ambient (green), 0.0000021 V nm_rms_^−1^ (yellow), 0.0021 V nm_rms_^−1^ (blue) and 0.21 V nm_rms_^−1^ (red) electric field strengths.

In addition, the interactions of the phospholipid head groups with solvent and the dissolved charged ionic species, in particular, play an important role in the structural integrity of the bilayer.^38^ The mass density distribution identifying the individual system components provided insight into the placement of water molecules and ions relative to the POPC lipid membrane. Fig. 5A shows there are no appreciable differences between the membrane and water mass density distributions at ambient condition and under the external electric field. There is, however, a notable increase in sodium (Na^+^) ion concentration within the membrane leaflets appearing in the simulations of high strength (0.21 V nm_rms_^−1^) field, likely driven by the electrostatic interactions with the phosphate in the lipid head group enhanced by the electric field force. Studies have shown that sodium ions exhibit a strong interaction with the carbonyl oxygens of the lipids, forming tight ion–lipid complexes deep in the POPC membrane. This interaction is evidently enhanced by the presence of the electric field, possibly due to the increased lipid dynamics at high intensity electric fields, which allowed for higher concentration of sodium within the membrane. The resulting charge density is counterbalanced by a layer of chloride ions which remain within the water phase, as observed in ambient conditions as well.^37^

Radial Distribution Function (RDF) of molecular water (and individual oxygen and hydrogen atoms within the water molecules) relative to the phosphorous atom in the lipid phosphate groups were calculated to investigate the structuring of water around the membrane surface. Fig. 5C and D illustrates two hydration layers present at ∼3.7 Å and ∼6 Å separation from the membrane head group surface, with an average of 6.2 and 18.8 water molecules surrounding the phosphorous of the phosphate groups, respectively. Within the first hydration layer the water hydrogens are pointing towards the P atoms as the inset shows. Notably, there is no appreciable difference in the water location probability near the phosphate group for different applied field strengths.

**Fig. 6:**
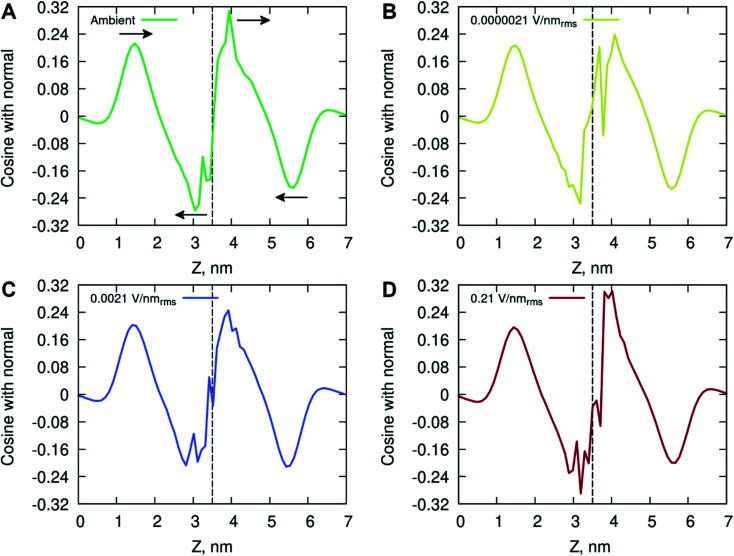
Average water dipole moment along the membrane normal at (A) ambient (green), (B) 0.0000021 V nm_rms_^−1^ (yellow), (C) 0.0021 V nm_rms_^−1^ (blue) and (D) 0.21 V nm_rms_^−1^ (red) electric field strength condition. Arrows in plot A indicate the direction of the water molecules orientation around the nearest extrema. The vertical line at ∼3.5 nm depicts the middle point of the bilayer.

The ordering of water in the interfacial region was further investigated, with results in Fig. 6 displaying the mean dipole moment of water molecules relative to their position within the lipid bilayer. Water molecules, having a large permanent dipole moment, exhibit substantial rotational motion in the presence of external electric fields. The plots identify a positive water dipole moment pointing into the center of the membrane, in the direction opposite to that of the lipid dipoles, within the interfacial region of the membrane compartments. There is a gradual reorientation of the water dipole moment as the water molecules move deeper into the bilayer possibly caused by the increased lipid dynamics and water–lipid head hydrogen bonding (discussed below). The large fluctuations inside the hydrophobic core of the lipid bilayer are likely due to individual water molecules reorientating while permeating the membrane. These fluctuations are more pronounced for the membrane system exposed to high electric field strength (0.21 V nm_rms_^−1^).

The effects of high frequency electric fields on the hydrogen bonding of water to the POPC membrane was determined by calculating the average number of hydrogen bonds formed and their lifetimes with the different lipid groups. A hydrogen bond was defined by a geometrical criterion with a maximum donor–acceptor distance of 0.35 nm and a donor-hydrogen-acceptor angle of 30°, whereby the OH and NH groups were regarded as donors and the O and N atoms as acceptors.

The results in Table 1 demonstrate the deep penetration of water in the POPC membrane up to the acyl carbonyl groups. Specifically, the oxygens of the phosphate group were most engaged in hydrogen bonding due to their extensive contacts with water, followed by the carbonyl atoms of the acyl tails. It is evident that the ester oxygen atoms have a lower probability to form hydrogen bonds than carbonyl oxygen atoms, as also demonstrated by others.^39,40^ This is indicated by the smaller number of hydrogen bonds formed with the glycerol groups (Table 1) and the O11 and O12 atoms compared to the O13 and O14 atoms of the phosphate group (Table 2), each of which had on average ∼0.6 hydrogen bonds to water *versus* ∼2.0 hydrogen bonds to water for the non-ester oxygens. While the hydrogen bonding behavior is consistent with numerous experimental and computational studies summarized in the review^41^ and others,^38,42,43^ there are some notable differences caused by the exposure to high energy electric fields. The results showed an increase in average number of hydrogen bonds formed between water and the POPC membrane in presence of high intensity electric fields (Table 1). This is evidenced by an increase in hydrogen bonding across all lipid head groups, indicating persistent presence of water inside the membrane compartment. Interestingly, there is a reduction in the hydrogen bond lifetimes of the waters that are interacting with the oxygens in the glycerol and acyl tails suggesting there is increased water mobility causing vibrations and the faster disruption of the hydrogen bonds inside the membrane. In the context of solvation of proteins exposed to electric fields showed hydrogen bond dynamics experience substantial localised changes, depending on the water interactions with specific local residues and surface topology.^44^ The amplified local translational and rotational motion by charged and dipolar residues exposed to electric fields promoted increased kinetics (hydrogen-bond breakage and re-formation) in the protein-water hydrogen bond network. Other studies of water in EM fields, showed field-induced dipolar rotational motion of water molecules led to an increase in hydrogen-bond kinetics and self-diffusivity *via* roto-translational coupling.^45–47^

**Table 1:** Hydrogen bond analysis of the water–lipid interactions

	Average number of hydrogen bonds (HB_aver_) and hydrogen bond lifetimes (HB_life_)										
	Whole system			Choline		Phosphate		Glycerol		C=O of Acyl chains	
	HB_aver_	HB per lipid	HB_life_ (ps)	HB_aver_	HB_life_ (ps)	HB_aver_	HB_life_ (ps)	HB_aver_	HB_life_ (ps)	HB_aver_	HB_life_ (ps)
Ambient	2850.26 ± 29.34	7.13 ± 0.07	43.63	0.01 ± 0.11	—a	2126.05 ± 22.77	37.81	64.29 ± 7.79	20.38	679.90 ± 15.95	81.82
0.0000021 V nm_rms_^−1^	2857.51 ± 30.47	7.14 ± 0.08	48.21	0.01 ± 0.13	—	2129.10 ± 22.88	37.82	64.76 ± 7.54	21.43	663.63 ± 16.52	81.51
0.0021 V nm_rms_^−1^	2854.80 ± 32.33	7.14 ± 0.08	47.44	0.01 ± 0.13	—	2127.75 ± 22.74	42.25	65.76 ± 7.55	20.52	661.26 ± 18.11	80.99
0.21 V nm_rms_^−1^	2877.10 ± 28.56	7.19 ± 0.07	46.16	0.01 ± 0.13	—	2138.19 ± 22.08	40.05	66.74 ± 7.78	17.93	672.15 ± 15.60	74.74

a A dash (—) indicates no lifetime was determined due to the low number of hydrogen bonds.

**Table 2:** Average number of water hydrogen bonds formed with individual oxygens in the phosphate lipid group

	Hydrogen bonding with phosphate oxygens			
	O11	O12	O13	014
Ambient	228.65 ± 11.32	273.78 ± 9.82	810.80 ± 12.77	812.82 ± 13.00
0.0000021 V nm_rms_^−1^	228.84 ± 10.91	274.56 ± 10.07	811.09 ± 13.11	814.60 ± 13.21
0.0021 V nm_rms_^−1^	229.12 ± 11.16	274.12 ± 10.19	810.00 ± 13.30	814.49 ± 13.11
0.21 V nm_rms_^−1^	230.94 ± 10.53	276.85 ± 10.25	812.64 ± 12.81	817.75 ± 13.18

**Fig. 7:**
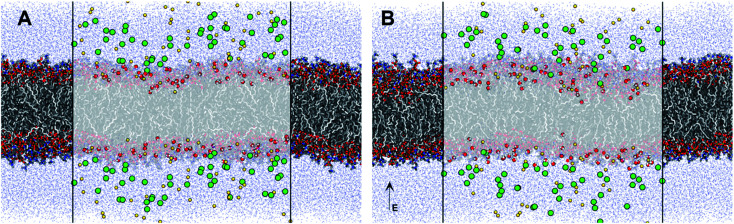
Molecular representation of the POPC membrane (shown in licorice) exposed to (A) ambient and (B) 0.21 V nm_rms_^−1^ high frequency electric fields. Water molecules within 1.6 Å of the membrane are shown in vdW surface representation, and all other water molecules as blue points. The sodium and chloride ions are shown as their vdW surfaces coloured yellow and green respectively.

The increased concentration of sodium ions, water hydrogen bonding and dipole fluctuations within the POPC bilayer appear to be induced by the exposure to electric fields of high strength (0.21 V nm_rms_^−1^) (Fig. 6 and 7). These observations provide direct atomistic insights into the molecular level events underpinning experimental findings showing that the high energy EMFs facilitate the penetration of hydrophilic nanoparticles without significant structural changes to the lipid bilayer, and stipulate the rationale presented below for the origins of these effects.

In order to understand the mechanism by which EMF exposure induced translocation of nanospheres across lipid bilayers, the likely events that take place under microwave were considered. The length of the wave produced by 18 GHz electromagnetic field is comparable to the typical diameter of bacterial cells.^2–4^ This means that the oscillation induced as a result of a transfer of energy from a polarized electromagnetic oscillation to molecules in ground state with cell suspensions will be most pronounced for small molecules, such as electrically charged ions and polar water molecules.^48^ Larger, bound molecules, such as those of the lipid bilayer, will also absorb some of the electromagnetic radiation, yet the magnitude of oscillations induced by such a transfer of energy may be significantly smaller and difficult to quantify. The EMF-induced oscillations, *i*.*e*., vibrations, of water molecules have the potential to induce bilayer permeability of liposomes without affecting their integrity, as demonstrated in this experiment.

Indeed, the mechanical vibration of water dipoles during EMF exposure couple with partial reorientation of alignment of water molecules under the effect of electric field leads to bending and/or breakage of hydrogen bonds, as shown by the MD data (Fig. 7 and Table 1). In turn, the vibrational motion-induced breakage of hydrogen bonds by water molecules may lead to changes in physicochemical (structural) properties of EMF-irradiated water, particularly with respect to gas solubility (by affecting the interface between liquid and gas, *e*.*g*., dissolved CO_2_, phases) and hydration level of water dissolved ions. As such, EMF irradiation can lead to formation of submicro- and nanometer sized (∼100 nm) bubbles filled with several hundred or less gas molecules,^49^ though these effects are not observable in the all-atom simulations performed herein due to a much larger length scale of the phenomenon.

The hydration shells surrounding thus-formed bubbles can prompt interfacial ordering of water, with the latter becoming a potentially prominent perturbation mechanism in solution, as shown in material synthesis applications.^50^ These bubbles are relatively stable, and may interact with other molecules to form large complexes or supramolecular structures in colloidal suspensions.^51^ It has been suggested that the inherent presence of such bubbles and their subsequent bridging on the surfaces of hydrophobic materials in suspensions may be responsible for their long-ranged attraction.^49^ EMF exposure is thought to facilitate bubble nucleation by decreasing gas solubility, with the concentration of bubbles increasing as a function of increasing treatment time, working power and the initial dissolved oxygen concentration.^52^ The collapse of thus-formed bubbles may also lead to localised sharp increase in temperature,^53^ with the latter having capacity to catalyse chemical reactions, such as hydrolysis of molecules comprising lipid bilayers.^54^

The permeation process described in this study differs from electroporation which is another technique used in breaching the cell membrane barrier^55,56^ which results in increasing cell membrane permeability.^57^ Electroporation can be applied to release nucleic acids, intracellular proteins and other metabolites out of the cell for analysis purposes.^55^ The mechanism behind electroporation is the increase in transmembrane potential (TMP) of a cell above 0.2–1 V,^55^ irrespective of the cell type leading to reversible pore formation. These electro-pores allow DNA or large molecules to enter the cell^55–60^ by the application of external pulsed electric fields (PEFs).^61^ The method uses pulsed electric field to cause permeability of cell membranes.^55^ The application of an external pulsed electric field (PEF) of adequate strength and duration helps to obtain the threshold.^58^ Therapies based on electroporation involved placing electrodes around or within a target tissue while delivering a series of 8–100 short (∼100 μs) electric pulses of high voltage (∼1000–3000 V);^58^ the membrane can return to its intact state after pulse withdrawal.^55,56,62^ Cell membrane permeabilization resulting from the exposure to the EMF of 18 GHz can be classified as a different phenomenon compared to cell poration phenomena achieved using other techniques, including mechanical stress, sonoporation, electroporation and photoporation.^57,60,63^

An EMF-induced change in the hydration of H_3_O^+^ and OH^−^ typically present in water, specifically the removal of the hydration shell, may increase their chemical activity,^64,65^ leading to solution structuring, *e*.*g*., cluster formation, and reactive species production,^66^ as demonstrated by retained activity of the solution after EMF is removed.^67^ It has been shown that the size of the clusters detected in EMF-treated water was smaller than that of control, at 5–6 *versus* 10–13 molecules, respectively, resulting in a higher rate of dissolution of silica nanoparticles in the former.^65^ The effect of magnetic field on adsorption and desorption of water molecules has also been shown to be affected by their location, with water molecules condensed in pores, in multilayers, and in clusters surrounding hydrophobic functional groups on surfaces responding to magnetic fields more that molecules in the first layer on hydrophilic surfaces.^68,69^

The vibration-induced formation of biochemically reactive species may include the following reactions:(H_2_O)_*n*_(H_2_O ← H–OH → OH_2_)(H_2_O)_*m*_ → (H_2_O)_*n*_(H_2_O + H˙ + ˙OH + OH_2_)(H_2_O)_*m*_2˙OH → H_2_O_2_^3^O_2_ + ˙H → HO_2_˙HO_2_˙ + ˙H → H_2_O_2_˙OH + H_2_O_2_ → HO_2_˙ + H_2_OHO_2_˙ + HO_2_˙ → H_2_O_2_ + ^1^O_2_*hν* + H_2_O_2_ → 2 ˙OH

Thus-generated reactive oxygen species may engage into chemical reactions with macromolecules comprising the lipid bilayer of the GUVs. Therefore, in addition to the reorientation of the water molecules and ionic species at the membrane interface as shown by our modelling and the enhanced poration due to molecular vibrations, EMF exposure has the potential to induce chemical and thus further conformational changes in phospholipids comprising the bilayer.

The changes were in part attributed to hydrolysis of carboxylic and phosphoric esters of the lipids by hydrogen peroxide formed in water during EMF treatment. An EMF-induced change in lipid chemistry and packing affected the curvature of the lipid planes^70^ and membrane hydration,^71^ in line with the thinning and increased lipid lateral mobility (diffusion) of the lipids in the intermediate motional regime, were shown to increase the permeability of the lipid bilayer.^72^ The membrane curvature has also been shown to affect the manner in which other molecules bind to its surface.^73^

Another EMF effect on matter that warrants discussion is the possibility that the EMF induces a local mechanical change, *i.e.* a local elastic tension in the lipid bilayer through Maxwell's tensor, which can prompt the liposome to elongate or flatten from its spherical shape.^74–76^ Commensurate with experimental findings, our modelling showed a decrease in membrane thickness at high electric field conditions. The interaction between a polarised electromagnetic oscillation and matter results in both energy and momentum transfer. Here, the momentum exchange involves both the induction of a mechanical wave through the medium (due to pulling by the field on the medium), and partial transfer of the electromagnetic momentum to the medium, to which the medium responds by adjusting the internal stress components, with the resulting perturbation having zero total momentum.^77^ However, in the case of liposomes treated with 18 GHz, no visible change in the shape of the liposome was observed.

## 4. Conclusions

The results obtained in this study provide the first evidence of translocation of silica nanospheres through giant unilamellar vesicles without compromising their integrity. Enhanced GUVs permeability was achieved after three cycles of exposure to high frequency electromagnetic fields of 18 GHz. Overall, the molecular dynamics simulations of POPC membrane exposed to high frequency electric fields of different field strengths showed extremely high electric field strength (0.21 V nm_rms_^−1^) required for any appreciable effects to be observed. Such high electric field strength simulations identified an increase in the average area per lipid, a decreased order in the hydrophobic core, a reduction in membrane thickness, an increase of cation presence within the bilayer and increased water fluctuations evidenced by dipole moment reorientation and increased hydrogen bonding and dynamics. The simulations of POPC membrane under ambient and lower strength (<0.21 V nm_rms_^−1^) electric field conditions showed no significant differences in the membrane thus indicating lack of direct effect, rather, the EMF-induced oscillations, *i*.*e*., vibrations and reorientation of water molecules (dipoles) may be able to induce bilayer permeability without affecting their integrity. Thus, in this work we present a conceptually novel mechanism of membrane permeabilization. The EMF-induced permeabilization demonstrated in this study can offer new opportunities in applications of EMF for delivering or transporting cargo materials (*e.g*., drugs) to and across live cell membranes.

## Conflicts of interest

There are no conflicts to declare.

## Supplementary Material
